# A simple method to take urethral sutures for neobladder reconstruction and radical prostatectomy

**DOI:** 10.4103/0970-1591.36729

**Published:** 2007

**Authors:** B. Satheesan, N. Kathiresan

**Affiliations:** Division of Genito-Urinary Oncology, Department of Surgical Oncology, Cancer Institute (WIA), Adyar, Chennai - 600 036, India

**Keywords:** Radical prostatectomy, urethral sutures, vesico-urethral anastamosis

## Abstract

For the reconstruction of urethra-vesical anastamosis after radical prostatectomy and for neobladder reconstruction, taking adequate sutures to include the urethral mucosa is vital. Due to the retraction of the urethra and unfriendly pelvis, the process of taking satisfactory urethral sutures may be laborious. Here, we describe a simple method by which we could overcome similar technical problems during surgery using Foley catheter as the guide for the suture.

Following radical prostatectomy and radical cystectomy, preservation of external urethral sphincter and proper procurement of urethral sutures are important for subsequent reconstruction of the urinary tract to have a good continence and leak-free anastamosis. In open surgery, these may be often difficult due to unfriendly pelvis and retracted urethra. Hence alternative methods have been described for vesico-urethral anastamosis..[1,2] Here we describe a simple technique to overcome the problem of taking adequate sutures to acquire excellent results. We have tried this technique in our cases of radical cystectomy with orthotopic neobladder reconstruction and difficult radical prostatectomy. We have done 12 orthotopic neobladders and three radical prostatectomies so far. For the last five cases of orthotopic neobladders and all cases of radical prostatectomies, we used the technique described below. The anatomical and functional results have been very satisfying.

## TECHNIQUE

**Figure 1:**
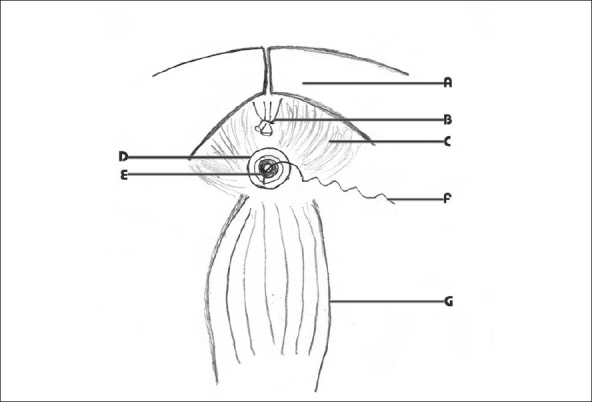
Half circle round-bodied needle is taken out through the urethra and Foley catheter A-Pubis, B-Dorsal venous complex, C-Levator ani, D-Urethra E-Divided Foley catheter F-Needle with the suture material G-Rectum

During cystectomy and radical prostatectomy, the urethra is divided anteriorly after the control of the dorsal venous plexus. The external sphincter extends almost up to the middle of the prostate. Hence the dorsal venous plexus is divided using curved Mayo's scissors at the middle of the prostate between ligatures and the scissors should move cutting the underlying tissue in an oblique direction up to the apex without violating the capsule. This leads to retraction of the prostatic extension of the external sphincter. When the urethro-prostatic junction is divided, the sphincter is undamaged. Thus the entire sphincter could be saved. It is required to be careful during radical prostatectomy as inadvertent rupture of the capsule should be avoided for oncological safety. Once the anterior wall of the urethra is divided, the Foley catheter is exposed. Using a long artery forceps, the catheter is pulled into the pelvis and the same is divided between two long artery forceps. Now there are two ends of the divided catheter in control—one on the urethral side and another on the bladder or prostate side. Once the resection of prostate or urinary bladder is performed, the urethral end of the Foley catheter is used as the guide for taking urethral sutures. Using atraumatic 2-0 monocryl suture with a half circle needle, a suture is taken at 3 O'clock position. The direction of the suture should be from outside to inside [Figures 1 and 3].

**Figure 2:**
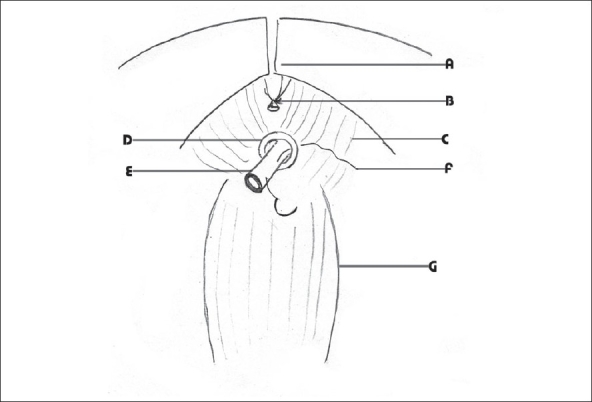
The divided Foley catheter is drawn out into the pelvis with the needle and the needle and thread can be separated from Foley catheter in retrograde fashion A-Pubis, B-Dorsal venous complex, C-Levator ani, D-Urethra E-Divided Foley catheter F-Needle with the suture material G-Rectum

**Figure 3:**
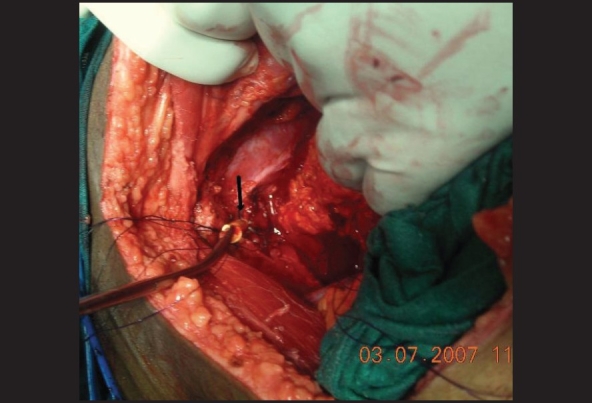
Half circle round-bodied needle is taken out through the urethra and Foley catheter

**Figure 4:**
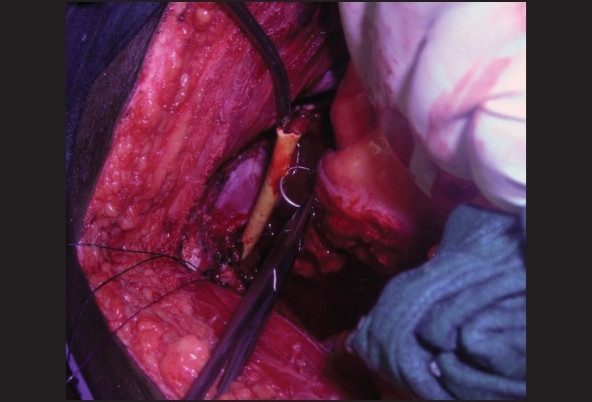
The divided Foley catheter is drawn out into the pelvis with the needle and the needle and thread can be separated from Foley catheter in retrograde fashion

While taking the suture, the cut end of the Foley catheter is withdrawn flush with the cut end of the urethra. The needle is passed through the urethra and the catheter. If the tip of the needle is visible, the same can be taken out through the catheter and the urethra. Then pull the catheter into the pelvis with the suture passing through it [Figures 2 and 4]. Now the needle could be tracked back through the catheter to free it from the catheter. We never had difficulty in retrieving the needle as the elasticity of the Foley catheter allows the return of the needle rather easily. In case difficulty arises the needle can be cut and a smaller-eyed needle could be used to thread the suture for the vesical suture. Thus the first urethral suture is taken. The advantage is that this method ensures adequate mucosa to be included in the suture. Similarly other sutures are taken one after the other till the number is adequate. If there is difficulty in taking the suture from the outside to inside direction, it is possible to take the sutures in the reverse direction. Here again the catheter end is kept flush with the urethral cut end and the suture is taken through the catheter first. Then pull the catheter into the urethra and the needle is passed through the urethra to the outside. The needle and the suture are retrieved. The catheter is pulled into the pelvis and then the suture is pulled out through the catheter between the catheter and the urethra. Thus inside to outside urethral suture could be taken. The major advantage we found with this technique is that even if the urethra gets retracted, adequate mucosa can be included in the suture.

## CONCLUSION

This is a simple technique using the Foley catheter to take urethral sutures for radical prostatectomy and neobladder reconstruction. Authors have experienced the ease of the procedure and satisfactory results.
